# Invertebrate Models of Dystonia

**DOI:** 10.2174/157015913804999504

**Published:** 2013-01

**Authors:** Kim A Caldwell, Yilong Shu, Nathan B Roberts, Guy A Caldwell, Janis M O’Donnell

**Affiliations:** Department of Biological Sciences, The University of Alabama, Tuscaloosa, AL 35487, USA

**Keywords:** *Drosophila*, *C. elegans*, torsinA, GTP-cyclohydrolase 1, Na+/K+ ATPase a3 subunit.

## Abstract

The neurological movement disorder dystonia is an umbrella term for a heterogeneous group of related conditions where at least 20 monogenic forms have been identified. Despite the substantial advances resulting from the identification of these loci, the function of many DYT gene products remains unclear. Comparative genomics using simple animal models to examine the evolutionarily conserved functional relationships with monogenic dystonias represents a rapid route toward a comprehensive understanding of these movement disorders. Current studies using the invertebrate animal models *Caenorhabditis elegans* and *Drosophila melanogaster* are uncovering cellular functions and mechanisms associated with mutant forms of the well-conserved gene products corresponding to DYT1, DYT5a, DYT5b, and DYT12 dystonias. Here we review recent findings from the invertebrate literature pertaining to molecular mechanisms of these gene products, torsinA, GTP cyclohydrolase I, tyrosine hydroxylase, and the alpha subunit of Na+/K ATPase, respectively. In each study, the application of powerful genetic tools developed over decades of intensive work with both of these invertebrate systems has led to mechanistic insights into these human disorders. These models are particularly amenable to large-scale genetic screens for modifiers or additional alleles, which are bolstering our understanding of the molecular functions associated with these gene products. Moreover, the use of invertebrate models for the evaluation of DYT genetic loci and their genetic interaction networks has predictive value and can provide a path forward for therapeutic intervention.

## INTRODUCTION

The complexity of the human nervous system represents a daunting challenge for researchers to attain a mechanistic understanding of the underlying cellular and molecular features responsible for disease onset and progression. The availability of a variety of invertebrate animal model systems is an essential component in the fight to combat movement disorders such as dystonia. No one model is ideal for every purpose and taking advantage of the specific attributes of different systems represents a prudent strategy to accelerate discovery. Here we outline the varied advantages and disadvantages of using different invertebrate models to inform and advance our understanding of dystonia.

Dystonia is an “umbrella” term used to define a series of clinically related disorders that can be categorized into subclasses, the primary and secondary dystonias. In both cases, involuntary muscle contractions force different parts of the body into abnormal movements and/or, often painful, postures [1]. Dystonic symptoms associated with secondary dystonias frequently coincide with other disease states such as Huntington's disease and juvenile Parkinson's disease. Additionally, medications (neuroleptics and calcium channel blockers) and toxins (carbon monoxide and wasp sting) can cause this form of dystonia. Conversely, primary dystonias have a hereditary component. In this regard, at least 20 genes are associated with monogenic dystonias, and most of these are inherited in an autosomal dominant manner with reduced penetrance. The gene products associated with nine different forms of dystonia have been identified. These gene products are torsinA (DYT1), TAF1 (DYT3), GTP-cyclohydrolase 1 (DYT5a), tyrosine hydroxylase (DYT5b), THAP1 (DYT6), myofibrillogenesis regulator 1 (DYT8), ε-sarcoglycan (DYT11), Na+/K+ ATPase a3 subunit (DYT12), PRKRA (DYT16), and SLC2A1 (DYT18) [2]. These findings provide the opportunity to develop animal models for *in vivo* evaluation of the encoded proteins, as well as identification of genetic and/or physical interactions. Nevertheless, this can be a daunting task, since screening for susceptibility factors, as well as the examination of cellular pathways, is labor intensive. 

Mammalian models are invaluable tools for examining the DYT gene products, especially considering the wide range of clinical features associated with dystonia, such as muscle contraction, tremors, and/or myoclonus [3-5], that may illustrate under-explored aspects of neuronal misregulation. Nevertheless, these models may not recapitulate all pathological impairments of dystonia and are expensive. In this regard, despite their lack of evolutionary complexity, invertebrate model organisms, such as *Drosophila melanogaster* and *Caenorhabditis elegans *have been utilized in first pass screens for examining cellular mechanisms, as well as identifying genes and drugs that might be therapeutically relevant to molecular processes underlying dystonia. These simple invertebrates share many conserved molecular pathways and cellular mechanisms with mammals, including homologs of DYT1, DYT5, and DYT12 (Table **1**). They also offer economical, yet strategic experimental paradigms that provide rapid analyses of pathological cellular mechanisms associated with dystonia. 

## STRENGTHS AND WEAKNESSES OF INVERTEBRATE MODELS

### A Caenorhabditis Elegans

The application of a limbless, microscopic soil nematode might not be the most overtly useful model for the investigation of human movement disorders. Yet, the distinct characteristics of the simple roundworm, *C. elegans*, have brought it to the forefront of biomedical research*. C. elegans* brings over 40 years of history to bear in terms of genetic mutants, anatomical information, and experimental methodology and is widely considered the best understood animal on Earth. Consisting of an invariable number of only 959 somatic cells, *C. elegans *affords unprecedented accuracy in the quantitation of cell and developmental deficits linked to genetic mutation. Concerted efforts by ambitious worm researchers throughout the 1970s and 80s led to both complete cell lineage and neuronal connectivity maps of this organism [6-8]. 

The nervous system of *C. elegans *consists of precisely 302 neurons, with all major classes of neurotransmitters represented within its defined neuronal subtypes [9]. The transparent anatomy of this nematode enables use of the jellyfish Green Fluorescent Protein (GFP) and its color variants to illuminate processes and cell bodies of distinct neuronal classes (i.e., the 26 GABAergic neurons of the animal or the 8 dopaminergic neurons in the hermaphrodite), as well as subcellular structures such as synaptic vesicles [10]. Aberrant neuronal function or degeneration can be linked to simple behavioral readouts in many cases. These range from subtle loss of mechanosensory deficits associated with dopamine (DA) neuron loss to complete paralysis induced by excess cholinergic stimulation. 

In 1998, *C. elegans* was also the first animal to have its genome sequence completed [11]. With over 50% of worm genes sharing a counterpart in mammals, correlates in mutant or transgenic nematode lines can reveal and, potentially, more rapidly unravel functional relationships underlying molecular mechanisms of disease. The capacity to cost-effectively and rapidly generate transgenic nematodes in a matter of days facilitates rapid evaluation of functional modifiers of neuronal activity and dysfunction. An efficient mechanism for cloning genes of interest into expression vectors is *via *Gateway recombinational cloning (Invitrogen). In this regard, a single entry vector containing the gene of interest can be cloned into multiple expression vectors. Gateway vectors for most worm open reading frames have been generated by the *C. elegans* research community and are commercially available (Thermo) [12]. Following creation of expression vectors, the production of multiple isogenic lines of transgenic nematodes can be typically accomplished within 6-8 weeks. Considering that *C. elegans* reproduce readily (200-300 offspring in 3 days), the availability of multiple and distinct transgenic lines (i.e., with different expression levels) allows for an unequivocal level of confidence when analyzing gene activity. Thus, experiments can be designed and conducted with hundreds of animals for each data point or condition. Further, a multitude of distinct experimental paradigms can be examined with high statistical power. 

Analysis of diverse pharmacological agents using *C. elegans* is an active area of research. For example, given the unmet need in understanding environmental contributors to diseases,* C. elegans* has been utilized in environmental toxicology studies for the evaluation of exposure to many substances, including uranium, methylmercury, cadmium, and ionic liquids [13-17]. *C. elegans* research has also been utilized as a first step in drug discovery screening. Examples of this include a drug-repurposing screen whereby anticonvulsants have been found to extend life span [18]. Other drug studies have revealed neuroprotective properties following exposure to acetaminophen [19]. Further, specific polyphenols extend lifespan [20, 21] and a new calcium channel antagonist has been identified [22]. 

* C. elegans *has long been utilized as an outstanding genetic system for the identification of suppressors and enhancers of phenotypes by traditional mutagenesis and screening. Among the more recent additions to the arsenal of methods available to worm researchers is the ability to conduct large or genomic scale screens for gene function by RNA interference (RNAi) [23]. This technique involves the epigenetic inactivation of specific gene products by introduction of double-stranded RNA (dsRNA) targeting discrete segments of gene sequence that subsequently triggers degradation of the corresponding endogenous mRNA transcript. Worms eat bacteria as their primary food source. *C. elegans* can therefore be fed transformed strains of *E. coli* that contain plasmid vectors designed to express individual dsRNAs targeting specific genes. Animals fed dsRNA-producing bacteria will exhibit phenotypes associated with gene knockdown either later in development or after the subsequent generation of fertilized eggs hatch from RNAi-treated hermaphrodite parents [24]. As libraries of bacteria capable of targeting nearly all the 20,000 genes comprising the worm genome are available at nominal cost, the efficiency by which this method is performed represents a significant advantage over other model systems and has established a new paradigm for large-scale reverse genetics analysis. 

Despite these numerous positive attributes, *C. elegans* is sometimes a less ideal choice as an animal model for several reasons. While they are indeed simple animals, worms are not cells. Thus, biochemical approaches commonly taken to quantitate protein or neurotransmitter levels in neuronal cultures or transfected cell lines are more challenging due to the need to separate out cell types from an intact multicellular organism. *C. elegans *is indeed anatomically small (1 mm as a complete adult) and this can also preclude the ability to discern intracellular localization of proteins without the expertise and time required for electron microscopy. Likewise, the thick cuticle surrounding the worm body presents an impediment to achieving consistent immunostaining. The simple nervous system of the worm obviously does not lend itself to more complex behaviors. Nonetheless, this has not ruled out the ability of *C. elegans* researchers to investigate neuronal networks and responses linked to learning paradigms [25-27]. Furthermore, pharmacological modulators of neuronal transmission have been applied to identify genes and pathways associated with neurodevelopment and neurotransmitter function and that are conserved with mammalian systems [9].

### B Drosophila

The fruit fly, *Drosophila melanogaster*, has been in constant use as a genetic model organism for over a century. Many of the same features that attracted early geneticists to this organism are no less attractive in the age of medical genomics research. The organism is small and can be easily and inexpensively maintained in very large numbers in the laboratory. It develops from embryo to reproductively mature adult in less than two weeks and, depending upon culture conditions, can live up to 60-80 days. A century of close scrutiny and genetic manipulation have resulted in a robust and exquisitely sensitive collection of genetic tools, including point mutations and gene disruptions in almost all known genes in the *Drosophila *genome and a highly efficient and powerful transgenesis system, based upon the endogenous *Drosophila *transposon, the P element. Transgenesis methods now include utilization of site-specific recombinases to insert sequences of interest in defined locations, and replacement gene targeting, though not as heavily utilized as gene knock-outs and knock-ins that are now common in mouse models, is increasingly efficient [28]. Importantly, most of the approximately 14,000 genes of *Drosophila* are conserved in vertebrates, and more than 75% of candidate human disease genes have *Drosophila *orthologs [29, 30]. This genetic similarity is coupled with extensive conservation of cellular pathways and mechanisms of regulation, conservation that can extend to parallel physiological defects when such pathways are altered by mutation or environmental toxins. Of particular interest in the context of neurological disorders is that the regulation of nervous system development and neurotransmitter systems controlling behavioral traits such as movement are conserved in *Drosophila *and can be easily and rapidly assayed. Despite extensive conservation in cellular and physiological mechanisms, the neural circuitry in the fruit fly is highly simplified, though far more complex than in *C. elegans. *For example, the adult *Drosophila *brain has about 200 dopaminergic neurons, as compared to eight in the worm, but they are arranged in stereotypical clusters that vary little in the number of neurons per cluster. This arrangement is advantageous in that early effects on neuronal morphology and synaptic development, as well as neuron loss are readily assessed in a whole nervous system context that includes a wide array of neuron and glial cell types. While neurochemical and biochemical analyses remain challenging in *C. elegans, *they are readily conducted in *Drosophila* [31-35]. These features have led to extensive application of the *Drosophila *model in human disease research. Its small size, however, means that it is far more difficult to directly monitor function in subregions of the CNS or to quantify synaptic release than in mammalian or cell culture models. Moreover, the lack of transparency, an attractive feature of *C. elegans,* means that analysis of the *Drosophila *CNS is more laborious than investigation of neuronal morphology and circuitry in the nematode. Despite the additional difficulty of tracing neural circuitry in the more complex *Drosophila *nervous system, an exciting development that promises to alleviate barriers to such studies is the creation of methods based on the mouse Brainbow strategy wherein recombinase-based fluorescence labeling of neuron subclasses can be used to distinguish and subdivide expression patterns [36, 37]. 

**Fig. (1):**
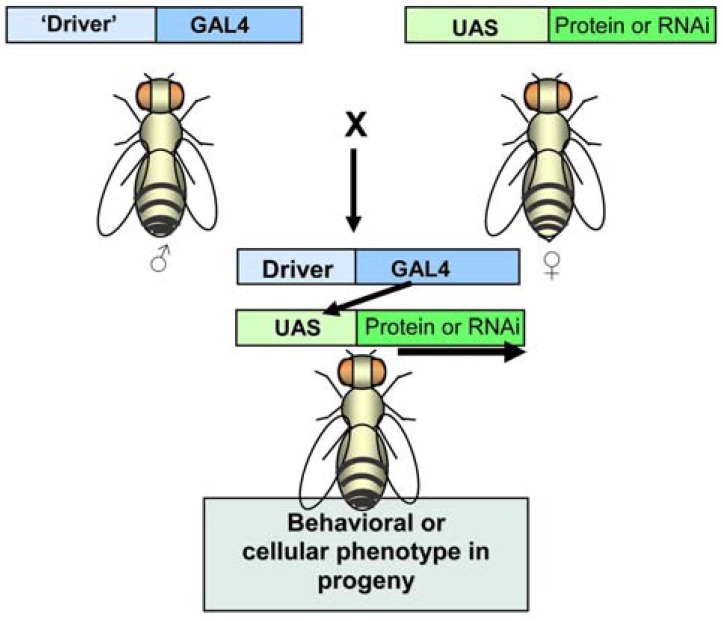
**The Gal4-UAS binary targeted gene expression system**. The system consists of a transgenic line in which coding sequence for the yeast transcription factor, GAL4, is under the control of a promoter or enhancer of interest and a second transgenic line in which the GAL4 target, Upstream Activating Sequence, controls transcription of a disease-related gene. When adults from the two strains are mated, the expression of the disease-related protein or RNAi is specifically targeted in the F_1_ progeny.

The challenge of conducting brain region-specific analyses in this small organism has been greatly ameliorated through the application of the powerful GAL4/UAS system for targeted transgene expression [38, 39]. GAL4 is a yeast transcription factor that activates the transcription of galactose-inducible genes in *Saccharomyces cerivisae*. The target sequences to which this transcription factor binds are termed Upstream Activating Sequences (UAS). Neither the gene encoding GAL4 nor the UAS elements are present normally in *Drosophila.* In this two-part system, one transgenic line is constructed in which the sequence to be expressed is under control of the UAS target. Expression, however, is typically quiescent until flies from this strain are mated to those of a second transgenic line in which GAL4 expression is controlled by a promoter element that is chosen to target a specific tissue type or one in which expression can be induced (Fig. **1**). Moreover, this system, when combined with expression of a temperature-sensitive version of *GAL80*, which is a *GAL4* repressor, allows for temporally-controlled induction of transgene expression.` This system has been employed extensively in several ways in movement disorder research. These include targeting expression of reporter genes, generally GFP, wild type or mutant *Drosophila *genes, or human disease genes. More recently, other binary and inducible expression systems have joined the arsenal of expression systems, bringing with them capabilities of manipulating the expression of multiple genes simultaneously and of individual genes with temporal and spatial precision [40]. 

While rapid and powerful large-scale RNAi screens are routine in *C. elegans *research*, *the more recent development of genome-wide *Drosophila *RNAi transgene libraries that utilize the binary expression strategy can now facilitate systems biology analyses of interacting gene networks [41, 42]. These tools have been particularly useful when expression occurs in the background of a mutation in a candidate disease gene such that, as in *C. elegans*, screens for enhancers or suppressors of a disease phenotype can be employed to detect previously unknown genetic contributors to disease mechanisms or to corroborate roles of candidate genes. In addition, toxins that trigger disease phenotypes can be administered simply by ingestion of the agent; direct brain lesioning is unnecessary. Similarly, potentially therapeutic molecules can be screened rapidly and inexpensively through ingestion. Examples of these applications are illustrated below in descriptions of *Drosophila *disease models.

## INVERTEBRATE DYSTONIA RESEARCH

The genomes of *C. elegans* and *Drosophila* contain homologs of three monogenic dystonias: DYT1, DYT5, and DYT12 (Table **1**). Using the invertebrate model systems, a cellular understanding of the associated gene products has been accelerated, as described below.

### A DYT1: Early-onset Torsion Dystonia

**Fig. (2):**
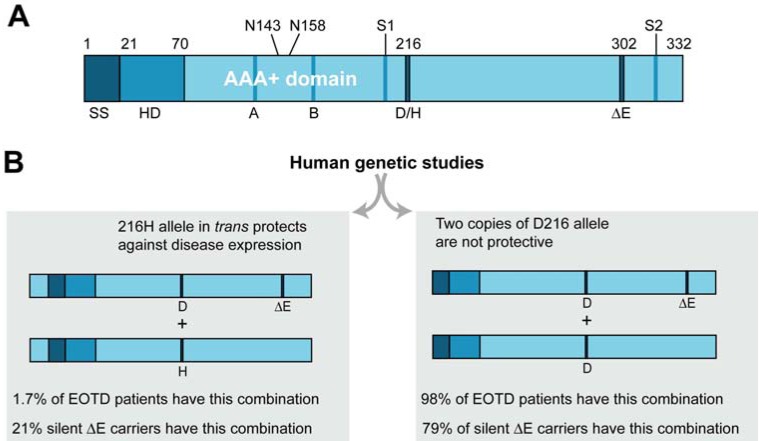
**Structural features of torsinA**. (**A**) TorsinA contains an ER signal sequence (SS) followed by the hydrophobic domain (HD), which is thought to localize torsinA to the ER. The AAA+ domain follows next with the Walker A motif (A), which binds ATP, and the Walker B motif (B) necessary for the hydrolysis of ATP. Two glycosylation sites (N143 and N158) are located between the Walker A and B motifs. Sensor I (S1) and Sensor II (S2) motifs are needed for sensing and hydrolyzing ATP as well. The torsinA (ΔE) deletion associated with most cases of EOTD is in the C-terminus. (**B**) Human genetic studies have determined that individuals with a D216H mutation in torsinA in trans with the (ΔE) mutation are far less likely to develop dystonia, whereby only 1.7% of EOTD patients will have this arrangement. In contrast, two copies of the D216 allele are not protective, as 98% of EOTD patients have this configuration.

Early-onset torsion dystonia (EOTD) is a rare neurological disorder characterized by sustained muscle contractions and abnormal posture. It is inherited in an autosomal dominant manner with onset beginning early in life [43]. Interestingly, this disease occurs with only 30-40% penetrance [44-46]. The primary alteration associated with EOTD, as found in most genetic cases, is a single glutamic acid deletion (ΔE) at position 302/303 in the C-terminus of a protein termed torsinA, occurring in only one allele of the *DYT1* gene (Fig. **2A**) [47]. 

Other very rare mutations have been identified in patients with EOTD, including an 18bp deletion in the C-terminus that does not appear to change torsinA localization and a codon change from arginine at position 288 to glutamine. Another individual was found to have a 4 base-pair deletion in *DYT1*, starting at position 312 of torsinA, although they appeared healthy and were not neurologically examined [48-50]. Yet another novel mutation in the conserved ATPase domain of torsinA (F205I) was identified in an individual with late-onset, focal dystonia; this is suggestive of a possible genetic link between inherited generalized and more common focal forms of dystonia [51]. Significantly, a polymorphism in torsinA at position 216, which changes an aspartic acid to a histidine, has been found at a higher frequency in healthy individuals who also harbor the (ΔE) mutation in the *trans* chromosomal configuration [52-54] (Fig. **2B**). This SNP has been suggested to result in a restoration of function from the (ΔE) mutation which is supported by a recent study that demonstrates a rescue of chaperone function *in vivo* when the D216H mutation was expressed in *trans* with (ΔE) torsinA but not in *cis* [55].

TorsinA is a member of the large and diverse AAA+ (ATPases Associated with Cellular Activity) family whose members have been found to participate in a variety of cellular mechanisms and pathways. These proteins all share the common AAA+ domain that is involved in ATP binding and hydrolysis, linking the energy of ATP to the mechanical work of the protein [47, 56-59]. Unlike any other AAA+ proteins, however, the subcellular location of all characterized torsin homologs is the lumen of the ER [60-63]. 

Similar to other AAA+ proteins, torsinA exists in a hexameric form held together by hydrophobic interactions [64]. The torsin family is comprised of four torsin genes in mammals consisting of torsinA, torsinB, torsin2, and torsin3, with several orthologs found in metazoans including zebrafish, *C. elegans* and *D. melanogaster* [65-67]. Sequence alignment revealed that all four mammalian torsin proteins contain an endoplasmic reticulum (ER) signal sequence and AAA+ domain and are localized to the ER lumen and nuclear envelope (NE) [67]. Although torsinB may be functionally similar, only torsinA exhibits high expression in neurons throughout the mammalian brain with subcellular localization extending from the ER and NE, to the cytoplasm and neuronal processes; this is suggestive of a neuronal specificity for torsinA expression that corresponds with its significance in EOTD [67-69].

The absence of the dystonia-associated glutamic acid (ΔE302) residue in torsinA results in the formation of aberrant membranous inclusions and redistribution of the protein to the nuclear envelope (NE) in neurons [70-72], thereby resulting in a net loss of native torsinA function at the ER. Human fibroblasts from *DYT1* patients also display a deficit for the processing of proteins through the secretory pathway [63]. Recent studies have implicated interactions between torsinA and cytoskeletal proteins termed nesprins that mediate connections between the NE and cytoskeleton [73].

#### i Application of C. elegans to DYT1 Dystonia Research

Initial studies in *C. elegans* on torsin-related proteins were focused on an embryonically expressed, ER-localized ortholog, termed OOC-5 (Table **1**). OOC-5 encodes a protein with 357 or 351 amino acids (dependent on alternative splicing). This protein has similar domain organization with human torsins and shares 40% sequence similarity [60]. Highlighting this similarity, OOC-5 is also a member of the AAA+ family [60, 74, 75] and studies of this protein might provide information regarding the activity of human torsinA. In this regard, the germline of *C. elegans* *ooc-5* mutants display an abnormal morphological appearance and produce smaller oocytes. The resulting fertilized embryos are also smaller (~60%-70% of the normal size) when compared to wild-type embryos. Notably, these embryos have defective cell polarization in which PAR proteins do not localize correctly, and the polarity defects are associated with changes in spindle orientation [60, 76]. PAR proteins are part of the conserved metazoan machinery utilized for polarizing cells [76]. Evidence from *in vitro* biochemical studies and transgenic nematodes expressing variants of OOC-5 revealed that redox changes associated with intrinsic disulfide bond formation appear to affect the binding of ATP and ADP and that mutants exhibit a low embryo hatch rate compared with control animals [77]. Specifically, two cysteine residues in the C-terminus were found to be critical for the function of OOC-5, which provides a possible explanation for the loss of torsinA function in DYT1 dystonia. It is therefore intriguing to speculate that the deletion of a single glutamate in the C-terminus of torsinA (ΔE) may alter the normal configuration of the protein, affect the formation of critical disulfide bonds in the C-terminus of torsinA, and thus ultimately affecting the normal function of this protein. 

**Fig. (3):**
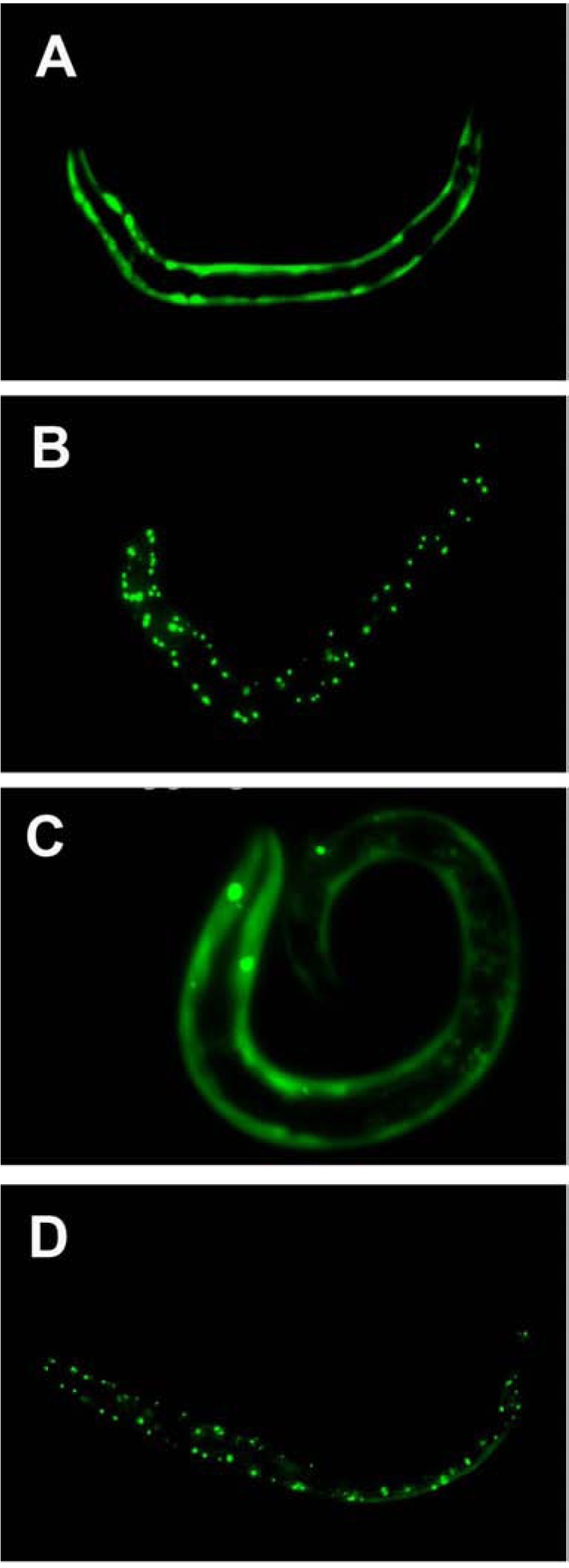
**Effects of *C. elegans* wild type or mutant TOR-2 on protein aggregation**. *In vivo* imaging of live transgenic worms demonstrates that strains of *C. elegans* containing integrated polyglutamine::GFP fusions of varying length exhibit soluble (**A**, **C**) or aggregated protein (**B**, **D**) in body wall muscle cells. (**A**) *C. elegans* expressing 19 glutamines fused to GFP (Q19::GFP) display soluble protein. (**B**) A worm expressing 82 glutamines fused to GFP have aggregated protein (Q82::GFP). (**C**) When worms co-express TOR-2 + Q82::GFP, the presence of wildtype TOR-2 reduces protein aggregation. (**D**) Conversely, Q82::GFP + TOR-2 (Δ368) worms do not display reduced levels of protein aggregation.

Native expression of the *C. elegans* torsinA ortholog TOR-2 (Table **1**) is limited to just a few cells of the hermaphrodite. These cells are the vulva muscle cells, a single cholinergic pharyngeal neuron (M1), two sensory neurons in the anterior region of the head (AW class), two interneurons of the head (AVE), and a few posterior neurons, including the pair of PVW neurons [78]. Additional studies with TOR-2 and human torsinA have demonstrated that they possess chaperone activity that is similar to that previously reported for other molecular chaperones. In this regard, overexpression of *C. elegans* TOR-2 or human torsinA can reduce protein aggregation resulting from polyglutamine repeats (Fig. **3A-C**) or α-synuclein *in vivo* [65, 79, 80]. Overexpression of human torsinA in H4 neuroglioma cells expressing α-synuclein also display significantly reduced protein aggregation [81]. Studies examining the *in vivo* chaperone activity of TOR-2 (Δ), the *C. elegans* mutant positionally equivalent to torsinA (ΔE), revealed that this mutation, as well as human torsinA (ΔE), no longer ameliorate polyglutamine aggregation (Fig. **3D**) [65, 80]. In a *C. elegans* model of DA neuron degeneration induced by a-synuclein, degeneration can be abrogated by overexpression of human torsinA or *C. elegans* TOR-2 but not torsinA (ΔE) [78]. 

*In vitro* biochemical evidence, obtained using bacterially expressed and purified human torsinA fusion proteins, has demonstrated that both wild type (WT) torsinA and mutant torsinA (ΔE) are capable of suppressing heat-induced aggregation of non-native misfolded proteins [82]. This suggests that both forms of torsinA retain chaperone activity and that the (ΔE) mutation does not result in a complete loss-of-function protein. While the maintenance of torsinA (ΔE) activity *in vitro* is in direct contrast to the loss of activity of torsinA (ΔE) observed *in vivo*, at least in terms of chaperone function, this may be a result of mislocalization of the mutant form to the NE, premature degradation, or possible entrapment to a substrate, rendering it unable to function or associate with other proteins. In combination with the previous studies, torsin homologs might have a role in the cellular management of protein misfolding. However, EOTD has not been characterized as a protein aggregation disease. The absence of neurodegeneration in dystonia suggests that more subtle causes of cellular dysfunction underlie the disease state. Moreover, the fact that only 30-40% of *DYT1* carriers display symptoms is further indication that disease penetrance is potentially an outcome of unknown environmental influences and/or cellular processes that are more stochastic in nature [83]. Taken together, functional studies in *C. elegans* imply a cellular role for torsinA as a redox-regulated protein with chaperone-like activity. These data correlate with a hypothesis whereby dystonia is a manifestation of a failure in a torsinA-mediated mechanism that would normally function to maintain homeostatic conditions of protein function in neurons.

**Fig. (4):**
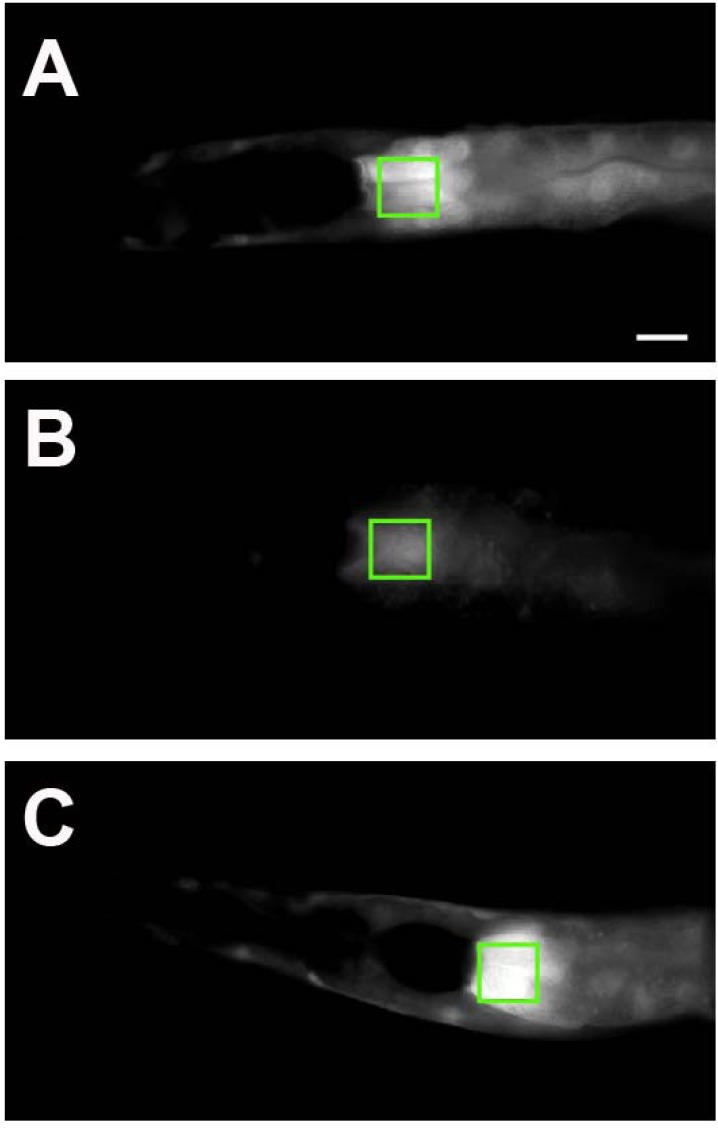
***C. elegans* ER stress response levels are mediated by the presence of torsinA.** (**A**-**C**) Transgenic nematodes carrying the ER stress reporter hsp-4::GFP were treated with tunicamycin (5 µg/ml; 5 hours) and then the amount of ER stress was measured in a 100 x 100 µm region just below the pharynx of all animals, as indicated in the white boxes. This is region consistently exhibits the highest levels of fluorescence intensity within the nematodes. (**A**) Worms expressing the ER stress response reporter hsp-4::GFP alone display easily observable fluorescence levels (that is, a high level of ER stress). (**B**) When human torsinA is co-expressed, the amount of fluorescence is greatly reduced (that is, much lower stress). (**C**) Mutant torsinA (ΔE) is not able to reduce ER stress as indicated by the high level of fluorescence. Scale bar = 100 µm.

In this regard, the impact of human torsinA variants on ER homeostasis and the unfolded protein response (UPR) was examined using transgenic nematodes [55]. WT torsinA-expressing worms attenuated ER stress induced by tunicamycin, however, worms expressing torsinA (ΔE) displayed an enhanced ER stress response (Fig. **4**). Furthermore, transgenic nematodes expressing both WT/ΔE demonstrated an ER stress response equivalent to ΔE alone, reflecting a dominant negative loss-of-function effect. Mouse embryonic fibroblasts devoid of torsinA (-/-) also showed enhanced ER stress [55]. Additional studies using transgenic nematodes expressing N-terminal torsinA truncations, revealed that only isoforms of torsinA predicted to localize within the ER demonstrated reduction in ER stress response. Human torsinA also associates with the ER-associated degradation (ERAD) pathway components Derlin-1, VIMP, and p97 [84]. Studies from mammalian cell lines demonstrated that torsinA is involved in the retrotranslocation and proteasomal degradation of improperly folded mutant cystic fibrosis transmembrane conductance regulator (CFTR) protein (CFTR ΔF508). Complementary studies in *C. elegans* demonstrated that ER stress associated with overexpression of CFTR ΔF508 was alleviated with overexpression of human torsinA [84]. Likewise, *DYT1* patient cells were more sensitive to ER stress and could not degrade CFTR ΔF508 are readily as control cells. While the example of CFTR is not directly relevant to dystonia, it is representative of normal torsinA function in maintaining the homeostatic balance at the ER. Therefore, it theoretically follows that neuron-specific membrane proteins (i.e. receptors, transporters) may be subject to the same torsinA-regulated mechanism, a process that is deficient in mutant torsinA-containing cells that contributes to the development or penetrance of EOTD.

*C. elegans* transgenic lines overexpressing human torsinA have also been used to investigate genetic modifiers of EOTD. Transgenic *C. elegans* that recapitulated a “protective” polymorphism identified in torsinA (D216H) were analyzed for changes in ER stress response [55]. As previously mentioned, human genetic analyses uncovered that torsinA (D216H), when carried in *trans *to the torsinA (ΔE) allele was “protective”; torsinA (D216H) was found at a higher frequency in non-manifesting DYT1 carriers and decreased in affected *DYT1* dystonia patients (Fig. **2B**) [53]. Transgenic worms overexpressing both torsinA (D216H) and torsinA (ΔE) exhibited an ER stress response that was equivalent to WT human torsinA while D216H alone or torsinA (ΔE) alone demonstrated a significantly increased ER stress response [55]. Therefore, these *C. elegans* assays provided a functional readout that reflected genetically relevant disease-modifying effects of dystonia-related mutations, illuminating a potential mechanism accounting for the reduced penetrance observed in EOTD.

Chemical modifiers of *DYT1* dystonia have also been investigated using transgenic lines of *C. elegans* overexpressing human torsinA. Using torsinA chaperone-like activity readouts in *C. elegans*, small molecules that selectively modulated the function of WT or mutant torsinA (ΔE) were identified [80]. Further analyses with one identified molecule, ampicillin, was performed in mammalian models. Strikingly, behavioral abnormalities in a *DYT1* knock-in mouse model were reversed following administration of ampicillin. Moreover, addition of this compound to human *DYT1* dystonia patient fibroblasts restored defective protein processing [80]. Ampicillin therefore selectively modifies torsinA activity in multiple models and represents a significant molecular lead toward novel treatment alternatives, even if antibiotics are not ideal for chronic dosing for dystonia. This study also demonstrates the expeditious and cost-effective nature of *C. elegans* as a predictive model in the translational path. 

#### ii Drosophila *Models of DYT1 Dystonia*

As in the *C. elegans *studies described above, an informative strategy for deciphering underlying mechanisms in DYT1 dystonia has been to utilize transgenic systems to express wild type and mutant forms of human torsin A in the *Drosophila *nervous system. In two such studies, the torsinA (ΔE) and torsinA (ΔF323-ΔY328) [torsinA (ΔFY)] mutant proteins as well as wild type torsinA were expressed pan-neuronally in larvae and adults, in dopaminergic and serotinergic neurons, and in presynaptic and post-synaptic neuromuscular junctions in the larval body wall using a variety of GAL4 drivers [85, 86]. The wild type and torsinA (ΔFY) mutant proteins exhibited diffuse cytoplasmic distribution, while the torsinA (ΔE) mutant protein aggregated around the NE and plasma membrane in a pattern reminiscent of the distribution in human cells. Though none of these proteins elicited locomotor abnormalities under standard culture conditions, exposure to a 38°C environment (standard culture conditions are 22-25°C) revealed the rapid onset of latent movement disorders, including convulsive leg and wing movements, when the mutant, but not wild type, forms of torsinA were expressed. This observation may be in line with second-hit genetic or environmental models [83] for explaining the variable penetrance of the mutant gene in humans. Abnormalities in the structure of NMJ synaptic bouton structures also were discovered when either of the two human mutant genes, but not the wild type gene, were expressed, along with apparent effects on Fascilin II, which functions in the maintenance of the synapse. In addition, interactions with the TGFβ signaling components were detected, suggesting that this avenue of investigation will be useful in identifying other genetic interactions contributing to the disorder. It should be noted that the synaptic structure abnormalities were triggered by the torsinA (ΔFY) mutant protein, as well as the torsinA (ΔE) protein, despite the apparently normal distribution of the torsinA (ΔFY) protein, indicating that this system can be a sensitive indicator of more subtle features of the disorder and that the movement abnormalities can be separated from the presence of torsin aggregates in this system

Other avenues of investigation have focused on the single *Drosophila *ortholog of human torsinA*,,* dtorsin (also known as torp4a). The *torsin/torp4a *gene in *Drosophila *encodes a product that is 34% identical to torsinA and of similar size (Table **1**). Initially, in the absence of mutant alleles of this gene, RNAi knockdown and over-expression of the wild type gene were used to assess the cellular functions of this torsin ortholog [87]. Expression was targeted to the *Drosophila *retina with the eye-specific GAL4 driver, *gmr-Gal4*. Despite the absence of detectable neurodegeneration in torsion dystonia patients, targeted dtorsin RNAi expression, in combination with a chromosomal deficiency that includes a deletion of the *dtorsin* gene led to retinal degeneration. Degeneration was accompanied by abnormal morphology of the pigment granules of the eye, which are lysosomal-related membrane-bound organelles. Interestingly, over-expression of wild type dtorsin in the eye protected against age-dependent retinal deterioration, potentially in line with proposed chaperone functions for torsinA, but also caused abnormalities in pigment granule organization. A screen for genes that enhanced the eye phenotype resulting from *dtorsin* overexpression made use of a series of chromosomal deletions that together span over 85% of the *Drosophila *genome. Subsequent testing of candidate genes lying within interacting segments detected homologs encoding proteins involved in cargo transport from Golgi to lysosomes. Others were predicted to interact with cytoskeletal components or oxidative stress scavenging pathways. Thus, the enhancers of torsin-related phenotypes identified in this screen provide evidence of cellular mechanisms shared with human torsinA. Interestingly, this screen also identified *Punch *(the fly homolog of human DYT5, encoding GTP cyclohydrolase I) as an enhancer of the torsin eye phenotype. This observation becomes more significant in the context of a recent study in which the effects of the knock-out of *dtorsin* *via *homologous recombination were investigated [88].

Utilizing a method known as “ends-out” gene targeting [89, 90], most of the coding sequence of the *dtorsin* gene was eliminated. The *torsin/torp4a* gene is located on the X-chromosome in *Drosophila. *Males lacking the *torsin* gene on their single X-chromosome survived to adulthood only rarely, and the few adult survivors were sterile (which made the generation of homozygous females impossible). Mutant males exhibited striking motor deficits and reduced pigmentation, while heterozygous females displayed no overt locomotion or pigmentation abnormalities. Because the mobility and pigmentation phenotypes were reminiscent of those displayed by DA-deficient mutants, further studies focused on exploring effects on dopaminergic pathways. Pan-neuronal or dopaminergic neuron, but not muscle-specific, expression of wild type *dtorsin* rescued the larval locomotion phenotype, as did ingestion of DA by mutant larvae. In contrast, ingestion of serotonin or octopamine, the functional equivalent of norepinephrine in *Drosophila *and other arthropods, had no effect on these phenotypes.

The observation that loss of *dtorsin *impacts dopaminergic function, coupled with the prior detection of interactions of this *dtorsin* with GTP cyclohydrolase, suggest an interesting linkage between the *Drosophila* homologs of DYT1 and DYT5. Similarly, numerous observations link mammalian torsinA function with DA regulation and signaling [91]. For example, transgenic mice expressing the human torsinA (ΔE) mutant form of torsinA pan-neuronally or specifically in midbrain dopaminergic neurons displayed defects in DA release [92, 93], while another transgenic mouse study revealed an increased in DA turnover in the striatum of mice expressing human torsinA (ΔE) [94]. 

**Fig. (5):**
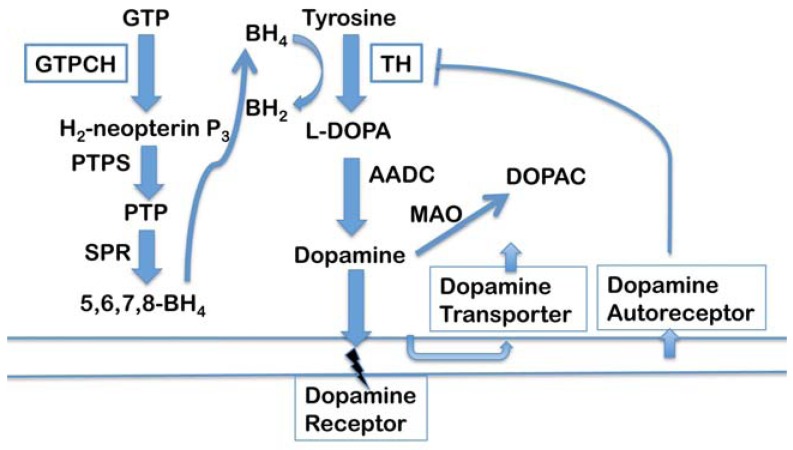
**Dopamine biosynthesis and transport**. Dopamine homeostasis requires complex and tightly coordinated biosynthesis and transport pathways in humans and in the *C. elegans* and Drosophila models. Participating proteins in worms and flies are conserved to varying extents, but all aspects of the biosynthesis and transport pathways, as well as homeostatic regulatory mechanisms are highly conserved. The synthesis of dopamine in presynaptic cells is rate-limited by two enzymes, tyrosine hydroxylase (TH) and GTP cyclohydrolase (GTPCH). TH converts tyrosine to L-DOPA with the assistance of regulating redox cofactor, 5,6,7,8-tetrahydrobiopterin (BH4), the synthesis of which is controlled by GTPCH. The remaining two enzymes in BH4 synthesis are 6-pyruvoyl tetrahydropterin synthase and sepiapterin reductase, while the final step in dopamine synthesis is catalyzed by aromatic amino acid decarboxylase. After synthesis, dopamine is packaged into synaptic vesicle *via* the vesicular monoamine transporter (not shown). Upon synaptic release, dopamine may interact with a post-synaptic dopamine receptor, be recycled back into the presynaptic cell *via* the dopamine transporter or may interact with the Dopamine D2 autoreceptor which triggers signaling events that result, amongst numerous responses, to the down-regulation of dopamine synthesis. Dopamine that remains in the cytosol is metabolized to dihydroxyphenyl acetate (DOPAC) by the enzyme monoamine oxidase.

Therefore, this investigation then turned to identifying components of the DA homeostatic network in *Drosophila* that might interact with torsinA, providing clues to the normal cellular roles of this protein in dopaminergic cells. Fig. (**5**) illustrates components of this network that could affect DA-dependent functions. Further analysis revealed that dopaminergic neurons were present in mutant brains and expressed tyrosine hydroxylase, the first and rate-limiting enzyme in dopamine biosynthesis. Thus, unlike RNAi knock-down of dtorsin in the fly eye [87], neural degeneration in torsin-deficient CNS was not evident. Nevertheless, DA pools in hemizygous males and heterozygous females were strongly reduced, which could be due to diminished synthesis of, or increased turnover of, this catecholamine. Although levels of the DA metabolite, 3,4-dihydroxyphenylacetic acid (DOPAC), which can be employed as an indicator of DA turnover, were not elevated, DOPAC:DA ratios were increased, as in some *DYT1* mouse models [94], suggesting possible trafficking abnormalities. Subsequent assays of tyrosine hydroxylase and immunoblotting of head extracts revealed no significant diminution of the synthesis or activity of this enzyme when supplied with substrate and its cofactor tetrahydrobiopterin (BH_4_) in *in vivo *assays. In contrast, the* dtorsin* mutants had severely depressed GTP cyclohydrolase activities and protein levels. This enzyme catalyzes the first and rate-limiting step in BH_4_ synthesis and is encoded by the gene *Punch* in *Drosophila *(Fig. **5**). Thus, these results support the earlier identification of *Punch*, as a d*torsin*-interacting gene [87]. Subsequent interaction experiments demonstrated that *torsin^-/+^; ple^-/+^* mutants, doubly heterozygous for dtorsin and tyrosine hydroxylase, respectively, displayed a mildly enhanced locomotion phenotype, while heterozygous *Punch *mutations strongly enhanced mobility deficits in combination with the heterozygous *torsin *knock-out mutation. Thus, torsin acts as a positive regulator of GTP cyclohydrolase (DYT5a) in *Drosophila. *While studies currently in progress are aimed at identifying the underlying molecular mechanism of this interaction, these results provide evidence of a normal cellular function for the torsin protein, as well as an intriguing functional connection between the genes associated with DYT1 and DYT5 dystonias. Studies are in progress to determine whether human torsinA interacts in a comparable manner with GTP cyclohydrolase and the DA homeostatic machinery. 

### B DYT5: DOPA-responsive Dystonia

DOPA-responsive dystonia (DRD), is a lower limb dystonia that exhibits diurnal fluctuation [95]. As the name of this disorder suggests, dystonic symptoms are alleviated by L-DOPA, the precursor of DA. In 1994, this dystonia was reported to be associated with dominantly inherited mutations in the gene encoding GTP cyclohydrolase I (*GCH*-*I*) [96], resulting in diminished availability of the BH_4_ cofactor, which is essential for catecholamine synthesis. Since this initial report, recessively inherited mutations in other genes in the cofactor pathway, as well as mutations in the gene encoding tyrosine hydroxylase also have been detected in some DRD families [97, 98]. Currently, the dominant *GCHI* form of dystonia is designated DYT5a, while recessive *TH *mutant forms comprise DYT5b. 

GCH-I is highly conserved in *Drosophila*, with catalytic domain sequences approaching 80% similarity and 70% identity (Table **1**). Dominant and recessive alleles of the *Drosophila GCH-1 *gene, *Punch, *have been generated [99, 100], and both endogenous and recombinant *Drosophila *GCH-1 (dGCH) have been purified and characterized kinetically [32, 101]. The kinetic and structural features of the *Drosophila *GCH-I closely parallel those of mammalian forms. Moreover, as in DRD, heterozygous *dGCH-I/Punch* mutants exhibit reduced levels of both the cofactor and DA in the CNS [31, 33]. Phosphorylation-dependent regulatory mechanisms have been described for dGCH [32], and of particular potential importance for modeling DRD, it has been shown that dGCH and tyrosine hydroxylase directly interact in a mutual regulatory mechanism that efficiently determines DA output [34]. In addition further regulatory interactions between *Drosophila* GCH-I and other proteins involved in DA homeostasis have been detected, suggesting additional levels of protein interaction complexity are likely in DRD [35]. Interestingly, it has been reported that a mouse model in which the gene encoding the second enzyme in BH_4_ synthesis (6-pyruvoyl tetrahydropterin synthase), was knocked-out, resulting in BH_4_ deficiency, displayed reduced tyrosine hydroxylase levels at nerve termini [102]. These observations emphasize the mechanistic links that are conserved between humans and *Drosophila. *These studies set the stage for further studies of interacting genes that modify dystonic symptoms. 

### C DYT12: Rapid-onset Dystonia-parkinsonism

Rapid-onset dystonia parkinsonism is a rare, autosomal dominant movement disorder. The symptoms, include both dystonic and parkinsonian features, with or without rigidity. There is a rapid progression (hours to weeks), but a maximum manifestation is reached very quickly. The gene product associated with DYT12 is the highly conserved alpha subunit of Na^+^/ K^+^ ATPase. It is a plasma membrane-localized sodium pump responsible for maintaining ionic gradients across cell membranes. The Na^+^/K^+^ ATPase pump is ubiquitously expressed in all animal cells [103] and is composed of alpha and beta subunits in invertebrate organisms, including *C. elegans* and *Drosophila*; vertebrates are predicted to contain an additional gamma subunit [104]. The alpha subunit is a catalytic subunit composed of ten transmembrane domains as well as an ATP-binding domain. The beta subunit is hypothesized to regulate processes such as the transport to, and insertion into, the membrane [105]. The loss of proper function of specific alpha subunits of Na^+^/K^+^ ATPase can result in two different human neurological diseases, familial hemiplegic migraine and DYT12 dystonia [106]. While the mechanism underlying these disorders remains undefined, studies in *C. elegans* and *Drosophila* have begun to provide clues to Na^+^/K^+^ ATPase function.

#### i *Studies of the Na^+^/K^+^ ATPase α3 Subunit in* C. elegans

The *C. elegans* Na^+^/ K^+^ ATPase α subunit, EAT-6 (Table **1**), was originally identified in a genetic screen for pharyngeal pumping (feeding) defects [107]. Mutations altering pharyngeal function are easily detected because the pharynx is responsible for ingesting food and ultimately, when it malfunctions, a starved phenotype results. Mutations in *eat-6* influence pharyngeal function, causing deficiencies in pharyngeal contraction and relaxation [107]. Further analysis revealed that human genomic sequences of the Na^+^/ K^+^ ATPase were capable of rescuing *eat-6* mutant phenotypes. Alterations to pharyngeal function caused by *eat-6* mutations are hypothesized to be a direct result of reduced Na^+^/K^+^ pump function in muscle cells. In addition to the pharynx,* C. elegans* EAT-6 is expressed in body-wall and vulval muscles, and at the neuromuscular junction (NMJ) along the ventral cord [108].

A role for the Na^+^/ K^+^ ATPase in synaptic regulation has also been revealed using mutant analyses [108]. In this regard, the localization and expression of nictotinic acetylcholine receptors (nAchRs) at the neuromuscular junction was altered in *C. elegans eat-6* mutants. The effects were allele specific; for example, a mutation within the large intracellular loop caused hypersensitivity to receptor agonists, while a mutation within the fifth transmembrane domain had no effect following exposure to synaptic receptor agonists. Furthermore, some alleles of *eat-6* alter the expression of nAchRs at postsynaptic NMJs and increase the formation of extrasynaptic nicotine-sensitive receptors in the NMJ [108]. Notably, based on the post-synaptic expression pattern of the Na^+^/ K^+^ ATPase subunits, as well as nAchRs, it is possible that EAT-6 may function as a scaffold protein for specific membrane-localized receptors. This interaction is not, however, directly associated with nAchR receptor subunits, as yeast-two hybrid studies failed to detect a direct interaction with EAT-6 [108]. At this time, the mechanism by which this protein controls the expression of nAchRs remains unknown. Additional studies have found that *eat-6* reduction *via *RNAi or EAT-6 overexpression caused hypersensitivity to the acetylcholinersterase inhibitor, aldicarb [109]. This suggests increased acetylcholine transmission with both reduced and enhanced levels of EAT-6. Furthermore, it was found that *eat-6* mutants are resistant to serotonin treatment, suggesting that EAT-6 is involved in the inhibitory modulation of acetylcholine by serotonin [109]. These studies indicate that the *C. elegans* homolog of the Na^+^/ K^+^ ATPase pump contributes to cellular dynamics at NMJs. Given the neuromuscular nature of dystonia, *C. elegans* *eat-6* models will most likely continue to reveal insights into the molecular nature of *DYT12* dystonia. 

#### ii *Studies of the Na^+^/K^+^ ATPase α3 Subunit in* Drosophila

The *Drosophila* model has also been effectively utilized to study Na^+^/ K^+^ ATPase activity *in vivo*. The *Drosophila* homolog of Na^+^/ K^+^ ATPase is *ATPalpha* (Table **1**). Mutations in *ATPalpha* cause deficiencies in locomotion, longevity, neurological dysfunction and neuronal degeneration [106]. An interesting aspect of *DYT12* in humans is the rapid onset of this disease following physical stress in patients. Notably, this stress-induced pathological feature is well recapitulated in *Drosophila* mutants where progressive locomotory impairment has been observed in *ATPalpha* mutant heterozygotes [106, 110]. Following mechanical stress, recovery is immediate in wild type controls, but *ATPalpha* mutants experience a delay, whereby temporarily paralysis is observed. This locomotory defect is progressive, with older animals displaying more paralysis [106]. While some *Drosophila* ATPalpha mutants show this phenotype following mechanical stress, others display temperature-dependent paralysis and reduced longevity [111]. A subset of ATPalpha missense mutants also exhibit vacuolar neuromuscular pathology changes throughout the brain [106, 111]. Two additional *ATPalpha* dominant temperature-sensitive (TS) paralytic mutations result in severe neurodegeneration. Young adult flies that express these TS mutant alleles display neuronal hyperexcitability before obvious neurodegeneration is detectable [111]. With these phenotypes, the *Drosophila* model has emerged as an excellent system for further examination of the neuronal defects resulting from Na^+^/ K^+^ ATPase mutations. 

## SUMMARY

Here we have outlined distinct facets of *C. elegans* and *Drosophila* models that have provided significant insights into how specific disease-related proteins contribute to the dysfunction associated with dystonia. Beyond this, the technological advances pioneered through model organism research, such as GFP, transgenic overexpression analyses, the Gal-4/UAS system, genetic screening, phenotype analysis, and drug screening have exponentially advanced our capacity to unravel mechanisms and define therapeutic targets for dystonia. However, this chapter only scratches the surface of the many contributions specific invertebrate models have made toward the investigation of dystonia. In the ongoing battle to combat movement disorders, the arsenal of animal models brought to bear in this fight has been steadily growing. While mouse models remain unequivocally central to dystonia research, zebrafish models of Parkinson’s disease, displaying spontaneous motor behaviors, have emerged [112]. The advantages of this genetically tractable vertebrate system are many, especially as applied to the investigation of neurodevelopmental aspects of movement disorders. Notably, the utility of zebrafish models is highlighted by their use in the identification of small molecule suppressors of polyglutamine toxicity [113, 114]. 

Finally, we would be remiss in not noting that continued use of cellular models, from yeast to mammalian cultures, remain vital in terms of gene and drug discovery, high-throughput screening, and target validation studies. The combined application and integration of animal and cellular models serves to confirm findings and accelerate the translational process of bringing new discoveries closer to the clinic.

## Figures and Tables

**Table 1.** Invertebrate Homologs of Monogenic Forms of Dystonia with Identified Gene Products

Dystonia Type	Gene Product	Drosophila Homolog	E-value	*C. elegans* Homologs	E-value
DYT1; early-onset torsion dystonia	torsinA	Torsin/torp4a	3e-67	TOR-1^a^ TOR-2 OOC-5	1.5e-56 6.4e-63 2.1e-57
DYT5a; Dopa-responsive dystonia (DRD)	GTP-cyclohydrolase 1	Punch	2e-98	CAT-4	6.4e-70
DYT5b; Dopa-responsive dystonia (DRD)	tyrosine hydroxylase	pale	1e-166	CAT-2	2.3e-97
DYT12; Rapid-onset dystonia-parkinsonism	Na+/K+ ATPase a 3 subunit	ATPa/Na pump a subunit	0.0	EAT-6	0

a possible pseudeogene; cDNA not confirmed or isolated by either academic or commercial labs.

## References

[1] Breakefield XO, Blood AJ, Li Y, Hallett M, Hanson PI, Standaert DG (2008). The pathophysiological basis of dystonias. Nat. Rev. Neurosci.

[2] Brüggemann N, Klein C (2010). Genetics of primary torsion dystonia. Curr. Neurol. Neurosci. Rep.

[3] Jinnah HA, Hess EJ (2008). Experimental therapeutics for dystonia. Neurotherapeutics.

[4] Tassone A, Sciamanna G, Bonsi P, Martella G, Pisani A (2011). Experimental models of dystonia. Int. Rev. Neurobiol.

[5] LeDoux MS (2011). Animal models of dystonia: Lessons from a mutant rat. Neurobiol. Dis.

[6] Sulston JE, Horvitz HR (1977). Post-embryonic cell lineages of the nematode. Caenorhabditis elegans Dev. Biol.

[7] Sulston JE, Schierenberg E, White JG, Thomson JN (1983). The embryonic cell lineage of the nematode *Caenorhabditis elegans*. Dev. Biol.

[8] White JG, Southgate E, Thomson JN, Brenner S (1986). The structure of the nervous system of the nematode *Caenorhabditis elegans. Phil. Trans. Royal Soc. London*. Series B Biol Scien.

[9] Bargmann CI (1998). Neurobiology of the *Caenorhabditis elegans* Genome. Science.

[10] Chalfie M, Tu Y, Euskirchen G, Ward WW, Prasher DC (1994). Green fluorescent protein as a marker for gene expression. Science.

[11] (1998). The *C. elegans* Sequencing Consortium: Genome sequence of the
nematode *Caenorhabditis elegans:* a platform for investigating
biology. Science.

[12] Lamesch P, Milstein S, Hao T, Rosenberg J, Li N, Sequerra R, Bosak S, Doucette-Stamm L, Vandenhaute J, Hill DE, Vidal M (2004). *C.elegans* ORFeome version 3.1: increasing the coverage
of ORFeome resources with improved gene predictions. Genome Res.

[13] Jiang GC, Hughes S, Stürzenbaum SR, Evje L, Syversen T, Aschner M (2009). *Caenorhabditis elegans* metallothioneins protect against toxicity induced by depleted uranium. Toxicol. Sci.

[14] Helmcke KJ, Syversen T, Miller DM, Aschner M (2009). Characterization of the effects of methylmercury on *Caenorhabditis elegans*. Toxicol. Appl. Pharmacol.

[15] Vanduyn N, Settivari R, Wong G, Nass R (2010). SKN-1/Nrf2 inhibits dopamine neuron degeneration in a *Caenorhabditis elegans* model of methylmercury toxicity. Toxicol. Sci.

[16] Cui Y, McBride SJ, Boyd WA, Alper S, Freedman JH (2007). Toxicogenomic analysis of *Caenorhabditis elegans* reveals novel genes and pathways involved in the resistance to cadmium toxicity. Genome Biol.

[17] Swatloski RP, Holbrey JD, Memon SB, Caldwell GA, Caldwell KA, Rogers RD (2004). Using *Caenorhabditis elegans* to probe toxicity of 1-alkyl-3-methylimidazolium chloride based ionic liquids. Chem. Comm.

[18] Evason K, Huang C, Yamben I, Covey DF, Kornfeld K (2005). Anticonvulsant medications extend worm life-span. Science.

[19] Locke CJ, Fox SA, Caldwell GA, Caldwell KA (2008). Acetaminophen attenuates dopamine neuron degeneration in animal models of Parkinson's disease. Neurosci. Lett.

[20] Abbas S, Wink M (2009). Epigallocatechin gallate from green tea (*Camellia sinensis*) increases lifespan and stress resistance in *Caenorhabditis elegans*. Planta Med.

[21] Saul N, Pietsch K, Stürzenbaum SR, Menzel R, Steinberg CE (2011). Diversity of polyphenol action in *Caenorhabditis elegans*: between toxicity and longevity. J. Nat. Prod.

[22] Kwok TC, Ricker N, Fraser R, Chan AW, Burns A, Stanley EF, McCourt P, Cutler SR, Roy PJ (2006). A small-molecule screen in C. elegans yields a new calcium channel
antagonist. Nature.

[23] Fire A, Xu S, Montgomery MK, Kostas SA, Driver SE, Mello CC (1998). Potent and specific genetic interference by double-stranded RNA in *Caenorhabditis elegans*. Nature.

[24] Kamath RS, Martinez-Campos M, Zipperlen P, Fraser AG, Ahringer J (2001). Effectiveness of specific RNA-mediated interference through ingested double-stranded RNA in *Caenorhabditis elegans*. Genome Biol.

[25] Hobert O, Carrera I, Stefanakis N (2010). The molecular and gene regulatory signature of a neuron. Trends Neurosci.

[26] Varshney LR, Chen BL, Paniagua E, Hall DH, Chklovskii DB (2011). Structural properties of the *Caenorhabditis elegans* neuronal network. PLoS Comput. Biol.

[27] Ardiel EL, Rankin CH (2010). An elegant mind: learning and memory in *Caenorhabditis elegans*. Learn. Mem.

[28] Venken JT, Bellen HJ (2012). Genome-wide manipulations of *Drosophila melanogaster* with transposons, Flp recombinase, and ?C31 integrase. Methods Mol. Biol.

[29] Reiter LT, Potocki L, Chien S, Gribskov M, Bier E (2001). A systematic analysis of human disease –associated gene sequences in *Drosophila melanogaster*. Genome Res.

[30] Doronkin S, Reiter LT (2008). *Drosophila* orthologues to human disease genes: an update on progress. Prog. Nucleic Acid Res. Mol. Biol.

[31] Krishnakumar S, Burton D, Rasco J, Chen X, O’Donnell J (2000). Functional interactions between GTP cyclohydrolase I and tyrosine hydroxylase in *Drosophila*. J. Neurogenet.

[32] Funderburk C D, Bowling K, Xu D, Huang Z, O’Donnell JM (2006). Atypical N-terminal extensions confer novel regulatory properties on GTP cyclohydrolase isoforms in *Drosophila melanogaster*. J. Biol. Chem.

[33] Chaudhuri A, Bowling K, Funderburk C, Inamdar A, O’Donnell JM (2007). Interaction of genetic and environmental factors in a *Drosophila* parkinsonism model. J. Neurosci.

[34] Bowling KM, Huang Z, Xu D, Funderburk CD, Karnik N, Ferdousy F, Neckameyer WS, O’Donnell JM (2008). Direct binding of GTP cyclohydrolase and tyrosine hydroxylase: Regulatory interactions between key enzymes in dopamine biosynthesis. J. Biol. Chem.

[35] Wang Z, Ferdousy F,  Lawal H, Huang Z, Daigle JG, Izevbaye I,  Doherty OM, Thomas J, Stathakis DG, O’Donnell JM (2011). Catecholamines up integrates dopamine synthesis and synaptic trafficking. J. Neurochem.

[36] Hampel S, Chung P, McKellar CE, Hall D, Looger LL, Simpson JH (2011). *Drosophila* Brainbow: a recombinase-based fluorescence labeling technique to subdivide neural expression patterns. Nat. Methods.

[37] Hadjieconomou D, Rotkopf S, Alexandre C, Bell DM, Dickson BJ, Salecker I (2011). Flybow: genetic multicolor cell labeling for neural circuit analysis in *Drosophila melanogaster*. Nat. Methods.

[38] Brand AH, Perrimon N (1993). Targeted gene expression as a means of altering cell fates and generating dominant phenotypes. Development.

[39] Duffy JB (2002). GAL4 system in *Drosophila:* A fly geneticist’s Swiss Army knife. Genesis.

[40] del Valle Rodríguez A, Didiano D, Desplan C (2011). Power tools for gene expression and clonal analysis in *Drosophila*. Nature Methods.

[41] Dietzl G, Chen D, Schnorrer F, Su KC, Barinova Y, Feliner M, Gasser B, Kinsey K, Oppel S, Scheiblauer S, Couto A, Marra V, Keleman K, Dickson BJ (2007). A genome-wide transgenic RNAi library for conditional gene inactivation in *Drosophila*. Nature.

[42] Flockhart IT, Booker M, Hu Y, McElvany B, Gilly Q, Mathey-Prevot B, Perrimon N, Mohr SE (2012). FlyRNAi.org-the
database of the *Drosophila* RNAi screening center: 2012 update. Nucleic Acids Res.

[43] Fahn S, Bressman SB, Marsden CD (1998). Classification of dystonia. Adv. Neurol.

[44] Bressman SB, de Leon D, Kramer PL, Ozelius LJ, Brin MF, Greene PE, Fahn S, Breakefield XO, Risch NJ (1994). Dystonia in Ashkenazi Jews: Clinical characterization of a founder mutation. Ann. Neurol.

[45] Klein C, Friedman J, Bressman S, Vieregge P, Brin MF, Pramstaller PP, De Leon D, Hagenah J, Sieberer M, Fleet C, Kiely R, Xin W, Breakefield XO, Ozelius LJ, Sims KB (1999). Genetic testing for early-onset torsion dystonia (DYT1): introduction of a simple screening method, experiences from testing of a large patient cohort, and ethical aspects. Genet. Test.

[46] Kamm C (2006). Early onset torsion dystonia (Oppenheim’s dystonia). Orphanet. J. Rare Dis.

[47] Ozelius LJ, Hewett JW, Page CE, Bressman SB, Kramer PL, Shalish C, de Leon D, Brin MF, Raymond D, Corey DP, Fahn S, Risch NJ, Buckler AJ, Gusella JF, Breakefield XO (1997). The early-onset torsion dystonia gene (DYT1) encodes an ATP-binding protein. Nat. Genet.

[48] Leung JC, Klein C, Friedman J, Vieregge P, Jacobs H, Doheny D, Kamm C, DeLeon D, Pramstaller PP, Penney JB, Eisengart M, Jankovic J, Gasser T, Bressman SB, Corey DP, Kramer P, Brin MF, Ozelius LJ, Breakefield XO (2001). Novel mutation in the TOR1A (*DYT1*) gene in atypical, early onset dystonia and polymorphisms in dystonia and early onset parkinsonism. Neurogenetics.

[49] Kabakci K, Hedrich K, Leung JC, Mitterer M, Vieregge P, Lencer R, Hagenah J, Garrels J, Witt K, Klostermann F, Svetel M, Friedman J, Kostic V, Bressman SB, Breakefield XO, Ozelius LJ, Pramstaller PP, Klein C (2004). Mutations in DYT1: extension of the phenotypic and mutational spectrum. Neurology.

[50] Zirn B, Grundmann K, Huppke P, Puthenparampil J, Wolburg H, Riess O, Müller U (2008). Novel TOR1A mutation
p.Arg288Gln in early-onset dystonia (DYT1). J. Neurol. Neurosurg. Psych.

[51] Calakos N, Patel VD, Gottron M, Wang G, Tran-Viet KN, Brewington D, Beyer JL, Steffens DC, Krishnan RR, Züchner S (2010). Functional evidence implicating a novel TOR1A mutation in idiopathic, late-onset focal dystonia. J. Med. Genet.

[52] Kock N, Naismith TV, Boston HE, Ozelius LJ, Corey DP, Breakefield XO, Hanson PI (2006). Effects of the genetic variations in the dystonia protein torsinA: identification of polymorphism at residue 216 as protein modifier. Hum. Mol. Gen.

[53] Risch NJ, Bressman SB, Senthil G, Ozelius LJ (2007). Intragenic Cis and Trans modification of genetic susceptibility in DYT1 torsion dystonia. Am. J. Hum. Genet.

[54] Kamm C, Fischer H, Garavaglia B, Kullmann S, Sharma M, Schrader C, Grundmann K, Klein C, Borggraefe I, Lobsien E, Kupsch A, Nardocci N, Gasser T (2008). Susceptibility to DYT1 dystonia in European patients is modified by the D216H polymorphism. Neurology.

[55] Chen P, Burdette AJ, Porter JC, Ricketts JC, Fox SA, Nery FC, Hewett JW, Berkowitz LA, Breakefield XO, Caldwell KA, Caldwell GA (2010). The early-onset torsion dystonia-associated protein, torsinA, is a homeostatic regulator of endoplasmic reticulum stress response. Hum. Mol. Genet.

[56] Weibezahn J, Bukau B, Mogk A (2004). Unscrambling an egg: protein disaggregation by AAA+ proteins. Microb. Cell Fact.

[57] Hanson PI, Whiteheart SW (2005). AAA+ proteins: have engine, will work. Nat. Rev. Mol. Cell Biol.

[58] Erzberger JP, Berger JM (2006). Evolutionary relationships and structural mechanisms of AAA+ proteins. Annu. Rev. Biophys. Biomol. Struct.

[59] White SR, Lauring B (2007). AAA+ ATPases: achieving diversity of function with conserved machinery. Traffic.

[60] Basham SE, Rose LS (2001). The *Caenorhabditis* elegans polarity gene ooc-5 encodes a torsin-related protein of the AAA+ ATPase superfamily. Development.

[61] Kustedjo K, Bracey MH, Cravatt BF (2000). Torsin A and its torsion dystonia-associated mutant forms are lumenal glycoproteins that exhibit distinct subcellular localizations. J. Biol. Chem.

[62] Liu Z, Zolkiewska A, Zolkiewski M (2003). Characterization of human torsinA and its dystonia-associated mutant form. Biochem. J.

[63] Hewett JW, Tannous B, Niland BP, Nery FC, Zeng J, Li Y, Breakefield XO (2007). Mutant torsinA interferes with protein processing through the secretory pathway in DYT1 dystonia cells. Proc. Natl. Acad. Sci. USA.

[64] Vander Heyden AB, Naismith TV, Snapp EL, Hodzic D, Hanson PI (2009). LULL1 retargets torsinA to the nuclear envelope revealing an activity that is impaired by the DYT1 dystonia mutation. Mol. Biol. Cell.

[65] Caldwell GA, Cao S, Sexton EG, Gelwix CC, Bevel JP, Caldwell KA (2003). Suppression of polyglutamine-induced protein aggregation in *Caenorhabditis elegans* by torsin proteins. Hum. Mol. Genet.

[66] Ozelius LJ, Page CE, Klein C, Hewett JW, Mineta M, Leung J, Shalish C, Bressman SB, de Leon D, Brin MF, Fahn S, Corey DP, Breakefield XO (1999). The TOR1A (DYT1) gene family and its role in early onset torsion dystonia. Genomics.

[67] Jungwirth M, Dear ML, Brown P, Holbrook K, Goodchild R (2010). Relative tissue expression of homologous torsinB correlates with the neuronal specific importance of DYT1 dystonia-associated torsinA. Hum. Mol. Genet.

[68] Augood SJ, Penney JB, Friberg IK, Breakefield XO, Young AB, Ozelius LJ, Standaert DG (1998). Expression of the early-onset torsion dystonia gene (DYT1) in human brain. Ann. Neurol.

[69] Konakova M, Pulst SM (2001). Immunocytochemical characterization of torsin proteins in mouse brain. Brain Res.

[70] Goodchild RE, Dauer WT (2004). Mislocalization to the nuclear envelope: an effect of the dystonia-causing torsinA mutation. Proc. Natl. Acad. Sci. U S A.

[71] Naismith TV, Heuser JE, Breakefield XO, Hanson PI (2004). TorsinA in the nuclear envelope. Proc. Natl. Acad. Sci. U S A.

[72] Torres GE, Sweeney AL, Beaulieu JM, Shashidharan P, Caron MG (2004). Effect of torsinA on membrane proteins reveals a loss of function and a dominant-negative phenotype of the dystonia-associated DeltaE-torsinA mutant. Proc. Natl. Acad. Sci. U.S.A.

[73] Nery FC, Zeng J, Niland BP, Hewett J, Farley J, Irimia D, Li Y, Wiche G, Sonnenberg A, Breakefield XO (2008). TorsinA binds the KASH domain of nesprins and participates in linkage between nuclear envelope and cytoskeleton. J. Cell Sci.

[74] Neuwald AF, Aravind L, Spouge JL, Koonin EV (1999). AAA+: A class of chaperone-like ATPases associated with the assembly, operation, and disassembly of protein complexes. Genome Res.

[75] Vale RD (2000). AAA proteins. Lords of the ring. J. Cell Biol.

[76] Basham S, Rose LS (1999). Mutations in *ooc-5* and *ooc-3* disrupt oocyte formation and the reestablishment of asymmetric PAR protein localization in two-cell *Caenorhabditis elegans* embryos. Dev Biol.

[77] Zhu L, Wrabl JO, Hayashi AP, Rose LS, Thomas PJ (2008). The torsin-family AAA+ protein OOC-5 contains a critical disulfide adjacent to Sensor-II that couples redox state to nucleotide binding. Mol. Biol. Cell.

[78] Cao S, Gelwix CC, Caldwell KA, Caldwell GA (2005). Torsin-mediated protection from cellular stress in the dopaminergic neurons of *Caenorhabditis elegans*. J. Neurosci.

[79] Hamamichi S, Rivas RN, Knight AL, Cao S, Caldwell KA, Caldwell GA (2008). Hypothesis-based RNAi screening identifies neuroprotective genes in a Parkinson's disease model. Proc. Natl. Acad. Sci. U S A.

[80] Cao S, Hewett JW, Yokoi F, Lu J, Buckley AC, Burdette AJ, Chen P, Nery FC, Li Y, Breakefield XO, Caldwell GA, Caldwell KA (2010). Chemical enhancement of torsinA function in cell and animal models of torsion dystonia. Dis. Model Mech.

[81] McLean PJ, Kawamata H, Shariff S, Hewett J, Sharma N, Ueda K, Breakefield XO, Hyman BT (2002). TorsinA and heat shock proteins act as molecular chaperones: suppression of alpha-synuclein aggregation. J. Neurochem.

[82] Burdette AJ, Churchill PF, Caldwell GA, Caldwell KA (2010). The early-onset torsion dystonia-associated protein, torsinA, displays molecular chaperone activity *in vitro*. Cell Stress Chaperones.

[83] Bressman SB, Fahn S, Hallet MA, De Long MR (2003). Dystonia genotypes, phenotypes and classification, in Dystonia 4: Advances in Neurology.

[84] Nery FC, Armata IA, Farley JE, Cho JA, Yaqub U, Chen P, da Hora CC, Wang Q, Tagaya M, Klein C, Tannous B, Caldwell KA, Caldwell GA, Lencer  I, Ye Y, Breakefield XO (2011). TorsinA participates in endoplasmic reticulum-associated degradation. Nat. Commun. doi: 10.1038/ncomms.

[85] Koh YH, Rehfeld K, Ganetzky B (2004). A *Drosophila* model of early onset torsion dystonia suggests impairment in TGF-ß signaling. Hum. Mol. Gen.

[86] Lee D-W, Seo JB, Ganetzky B, Koh Y-H (2009). ?FY mutation in human TorsinA induces locomotor disability and abberant synaptic structures in *Drosophila*. Mol. Cells.

[87] Muraro NI, Moffatt KG (2006). Down-regulation of torp4a, encoding the *Drosophila* homologue of torsin A, results in increased neuronal degeneration. J. Neurobiol.

[88] Wakabayashi-Ito N, Doherty OM, Moriyama H, Gusella J, O’Donnell JM, Ito N (2011). *dtorsin*, a *Drosophila* homolog of early-onset dystonia *DYT1*, plays a novel role in the dopamine metabolism. PLoS One.

[89] Gong WJ, Golic KG (2003). Ends-out, or replacement, gene targeting in *Drosophila*. Proc. Natl. Acad. Sci. USA.

[90] Gong WJ, Golic KG (2004). Genomic deletions of the *Drosophila melanogaster Hsp70* gene. Genetics.

[91] Wichmann T (2008). Commentary: Dopaminergic dysfunction in DYT1 dystonia. Exp. Neurol.

[92] Balcioglu A, Kim M-O, Sharma N, Cha J-H, Breakefield XO, Standaert DG (2007). Dopamine release is impaired in a mouse model of DYT1 dystonia. J. Neurochem.

[93] Page ME, Bao L, Andre P, Pelta-Heller J, Sluzas E, Gonzalez-Alegre P, Bogush A, Khan LE, Iacovitti L, Rice ME, Ehrlich ME (2010). Cell-autonomous alteration of dopaminergtic transmission by wild type and mutant (?E) TorsinA in transgenic mice. Neurobiol. Dis.

[94] Zhao Y, DeCuypere M, LeDoux MS (2008). Abnormal motor function and dopamine neurotransmission in DYT1 ?GAG transgenic mice. Exp. Neurol.

[95] Segawa M, Nomura Y, Nishiyama N (2003). Autosomal dominant guanosine triphosphate cyclohydrolase I deficiency (Segawa Disease). Ann. Neurol.

[96] Ichinose H, Ohye T, Takahashi E, Seki N, Hori T, Segawa M, Nomura Y, Endo K, Tanaka H, Tsuji S, Fujita K, Nagatsu T Hereditary progressive dystonia with marked kiurnal fluctuation caused by mutations in the GTP cyclohydrolase I gene. Nat. Genet.

[97] Lüdecke B, Dworniczak B, Bartholome K (1995). A point mutation in the tyrosine hydroxylase gene associated with Segawa’s syndrome. Hum. Genet.

[98] Knappskog PM, Flatmark T, Mallet J, Ludecke B, Bartholome K (1995). Recessively inherited L-DOPA-responsive dystonia caused by a point mutation (Q381K) in the tyrosine hydroxylase gene. Hum. Mol. Genet.

[99] Mackay W J, O’Donnell JM (1983). A genetic analysis of the pteridine biosynthetic enzyme, guanosine triphosphate cyclohydrolase, in *Drosophila melanogaster*. Genetics.

[100] Mackay WJ, Reynolds ER, O’Donnell JM (1985). Tissue specific and complex complementation patterns in the *Punch* locus of *Drosophila melanogaster*. Genetics.

[101] Weisberg EP, O’Donnell JM (1986). Purification and characterization of GTP cyclohydrolase I from *Drosophila melanogaster*. J. Biol. Chem.

[102] Sumi-Ichinose C, Ichinose H, Ikemoto K, Nomura T, Kondo K (2010). Advanced research on dopamine signaling to develop drugs for the treatment of mental disorders: Regulation of dopaminergic neural transmission by tyrosine hydroxylase protein at nerve terminals. J. Pharmacol. Sci.

[103] Kaplan JH (2002). Biochemistry of Na,K-ATPase. Annu. Rev. Biochem.

[104] Reeves AS, Collins JH, Schwartz A (1980). Isolation and characterization of (Na,K)-ATPase proteolipid. Biochem. Biophys. Res. Commun.

[105] Ackermann U, Geering K (1990). Mutual dependence of Na,K-ATPase alpha- and beta-subunits for correct posttranslational processing and intracellular transport. FEBS Lett.

[106] Ashmore LJ, Hrizo SL, Paul SM, Van Voorhies WA, Beitel GJ, Palladino MJ (2009). Novel mutations affecting the Na, K ATPase alpha model complex neurological diseases and implicate the sodium pump in increased longevity. Hum. Genet.

[107] Davis MW, Somerville D, Lee RY, Lockery S, very L, Fambrough DM (1995). Mutations in the *Caenorhabditis elegans* Na,K-ATPase alpha-subunit gene, *eat-6*, disrupt excitable cell function. J. Neurosci.

[108] Doi M, Iwasaki K (2008). Na+/K+ ATPase regulates the expression and localization of acetylcholine receptors in a pump activity-independent manner. Mol. Cell. Neurosci.

[109] Govorunova EG, Moussaif M, Kullyev A, Nguyen KC, McDonald TV, Hall DH, Sze JY (2010). A homolog of FHM2 is
involved in modulation of excitatory neurotransmission by
serotonin in *C. elegans*. PLoS One.

[110] Lebovitz RM, Takeyasu K, Fambrough DM (1989). Molecular characterization and expression of the (Na+ + K+)-ATPase alpha-subunit in *Drosophila melanogaster*. EMBO J.

[111] Palladino MJ, Bower JE, Kreber R, Ganetzky B (2003). Neural dysfunction and neurodegeneration in *Drosophila* Na+/K+ ATPase alpha subunit mutants. J. Neurosci.

[112] Bretaud S, MacRaild S, Ingham PW, Bandmann O (2011). The influence of the zebrafish genetic background on Parkinson's disease-related aspects. Zebrafish.

[113] Schiffer NW, Broadley SA, Hirschberger T, Tavan P, Kretzschmar HA, Giese A, Haass C, Hartl FU, Schmid B (2007). Identification of anti-prion compounds as efficient inhibitors of polyglutamine protein aggregation in a zebrafish model. J. Biol. Chem.

[114] Williams A, Sarkar S, Cuddon P, Ttofi EK, Saiki S, Siddiqi FH, Jahreiss L, Fleming A, Pask D, Goldsmith P, O'Kane CJ, Floto RA, Rubinsztein DC (2008). Novel targets for Huntington's disease in an mTOR-independent autophagy pathway. Nat. Chem. Biol.

